# Impact of Omicron BA.5 infection on maternal and neonatal outcomes

**DOI:** 10.3389/fcimb.2025.1551602

**Published:** 2025-05-02

**Authors:** Lu Li, Ruitian Hou, Zan Mai, Li Liang, Zheng Li, Bin Long, Lin Chen, Ping Feng, Baojun Yang, Lijie Yang, Lianhua Tang, Peizhi Wang, Fan Zhong, Mei Chu, Huichao Liang, Xiaoping Tang

**Affiliations:** ^1^ Institute of Infectious Diseases, Guangzhou Eighth People’s Hospital, Guangzhou Medical University, Guangzhou, China; ^2^ Department of Pathogenic Biology and Immunology, School of Basic Medical Sciences, Guangzhou Medical University, Guangzhou, China; ^3^ Guangzhou National Laboratory, Guangzhou, China; ^4^ Department of Obstetrics and Gynecology, Guangzhou Eighth People’s Hospital, Guangzhou Medical University, Guangzhou, China; ^5^ Department of Stomatology, Guangzhou Eighth People’s Hospital, Guangzhou Medical University, Guangzhou, China

**Keywords:** Omicron BA.5, pregnancy, neonate, vertical transmission, vaccination

## Abstract

**Introduction:**

Physiological and immunological adaptations during pregnancy may elevate the risk of adverse perinatal and neonatal outcomes associated with SARS-CoV-2 infection. This retrospective study aimed to explore the clinical characteristics of pregnant women and the maternal and neonatal outcomes during pregnancy following Omicron BA.5 variant infection.

**Methods:**

Clinical and laboratory data from 208 pregnant women with Omicron BA.5 infection were analyzed, including intrapartum and postpartum records of 24 infected parturients and their neonates, with comparisons made to uninfected controls. Multiple specimen types, including placental membranes and amniotic fluid, were collected for SARS-CoV-2 RNA detection.

**Results:**

Among 208 infected pregnant women, 91.8% (191/208) had received at least one dose of inactivated SARS-CoV-2 vaccine. BA.5 infection in pregnant women exhibited viral load, clearance time and symptom profiles comparable to the general population, with no severe or critical illness being found. No significant differences were noted between pregnant women over and under 35 years. BA.5 infection reduced the white blood cell counts but did not aggravate the hypercoagulability compared to the uninfected controls. Neonates of infected mothers showed a higher rate of intrauterine hypoxia than those controls. However, no SARS-CoV-2 RNA was detectable in any of the neonatal oropharyngeal swabs as well as maternal specimens, including placental membranes, amniotic fluid, vaginal secretions, breast milk, venous blood and ascites.

**Conclusion:**

This study demonstrates favorable maternal and neonatal outcomes in vaccinated pregnant women with BA.5 infection following timely medical intervention. Neonates born to infected mothers have an extremely low risk of vertical transmission. Nevertheless, enhanced prenatal care for pregnant women with COVID-19 remains essential to mitigate adverse neonatal outcomes.

## 1 Introduction

Coronavirus disease 2019 (COVID-19) continues to spread worldwide, with ongoing mutations of the SARS-CoV-2. Pregnant women, as a special group, have been a focal point of research due to the potential impact of COVID-19 on maternal and neonatal outcomes. In 2020, the World Health Organization assessed that SARS-CoV-2 infection during pregnancy is associated with an increased risk of intensive care for mothers, preterm birth, and admission for neonatal care for infants (Allotey et al., 2020). However, the effects of SARS-CoV-2 infection on pregnancy appear to vary depending on the viral variant (Birol Ilter et al., 2022). Emerging evidence indicates that the Delta variant is associated with more severe maternal and a heightened risk of stillbirth compared to the wildtype strain and Alpha variant (DeSisto et al., 2021). With the advent of the Omicron variant and widespread vaccination, the clinical manifestations and outcomes of COVID-19 have evolved. Recent studies suggest that Omicron variant appears to have lower pathogenicity among pregnant women compared to other variants (Lim et al., 2024). However, there have been reports indicating that Omicron infection is associated with increased postpartum hemorrhage and adverse neonatal outcomes (Li et al., 2023; Wang et al., 2024). To our knowledge, data on the specific impact of Omicron BA.5 subvariant infection during pregnancy remain limited, particularly in vaccinated populations. This study aimed to characterize the demographic and clinical profiles of pregnant women infected with Omicron BA.5, the majority of whom had received vaccine. Additionally, the study sought to systematically compare the perinatal and neonatal outcomes between pregnant women with and without COVID-19, thereby elucidating the potential impact of Omicron BA.5 variant infection on maternal and fetal health outcomes.

## 2 Materials and methods

### 2.1 Participants

A total of 604 patients from Guangzhou Eighth People’s Hospital, Guangzhou Medical University were divided into two groups: pregnant women with COVID-19 (n=225, hospitalized between October 1st to December 31st, 2022) and pregnant women without COVID-19 (n=379, hospitalized between January 1st to December 31st, 2020). 24 neonates born to SARS-CoV-2-infected mothers and 105 neonates from uninfected controls were included in the analytical cohort. Epidemiological evidence and viral genome sequencing demonstrated that COVID-19 individuals originated from a single Omicron BA.5 variant. This study was approved by Guangzhou Eighth People’s Hospital Ethics Committee (No. 2020C-K001). All patients provided their written informed consents.

### 2.2 Clinical and laboratory data collection

For both pregnant women cohorts, data on demographic, clinical characteristics and laboratory results were collected. The clinical characteristics include age, body mass index (BMI), Gestation at symptom onset, vaccination, clinical classification (asymptomatic, mild, moderate), comorbidities (hypertension, diabetes, psychosis, etc.) and symptoms (fever, cough, expectoration, etc.). Data regarding maternal conditions during delivery and neonatal outcomes were obtained from delivery and surgical records. The maternal outcomes included pregnancy risk, prenatal symptoms, delivery approaches (cesarean or vaginal delivery), delivery status (intrapartum hemorrhage, etc.), postpartum symptom and discharge status. Neonatal outcomes included gestational age at the time of delivery, birth weight, amniotic fluid, Apgar score and so on. The data were extracted and entered into the electronic database by two independent clinicians.

All diagnostic criteria were based on the National Health Commission’s Guidelines for Diagnosis and Treatment Protocol for COVID-19 (Trial version 9) (National Health Commission of the People’s Republic of China, 2022). Clinical characteristics included clinical classification (mild, moderate, severe), comorbidities (hypertension, diabetes, chronic heart disease, liver disease, lung disease, and thyroid) and symptoms (fever, cough, sputum, sore throat, dyspnea, vomit, headache, diarrhea, and fatigue). Demographic data included sex, age, birth history. Asymptomatic cases were diagnosed when patients showed no clinical symptoms but tested positive for SARS-CoV-2 viral RNA positive during the follow-up period after viral exposure or close contact with confirmed cases. Mild cases refer to those who had slight symptoms but without a sign of pneumonia on imaging. Moderate cases here refer to those who had a fever, and/or other respiratory symptoms with a sign of pneumonia on imaging. Severe cases refer to those who meet any of the following: (1) shortness of breath, respiratory rate≥30 times/min; (2) oxygen saturation ≤ 93% in resting state; (3) arterial partial pressure of oxygen (PaO2)/inhaled oxygen concentration (FiO2) ≤300mmHg (1mmHg=0.133kPa); and (4) progressively worsening of clinical symptoms and progression of pneumonia lesions>50% within 24 to 48 hours on imaging.

### 2.3 Specimen collection and viral RNA detection with RT-PCR

All clinical specimens were collected by well-trained medical staff at the same hospital, adhering strict standardized procedures. Nasopharyngeal or oropharyngeal swabs from pregnant women were collected regularly during hospitalization. Amniotic fluid, placental membranes, vaginal swabs and ascites specimens were collected from mothers with COVID-19 during delivery. The neonatal oropharyngeal swabs, the paired maternal breast milk and venous blood specimens were obtained within 24 hours postpartum. The specimens were stored in virus medium. Viral RNA was extracted within two hours using the Nucleic Acid Isolation Kit (Da’an Gene Co. Ltd, Cat: DA0630, China) according to the manufacturer’s instructions. Subsequently, RT-PCR was performed using the RNA Detection Kit for SARS-CoV-2 (Da’an Gene Co. Ltd, Cat: DA0930, China) (Hu et al., 2020; McAndrews et al., 2020). RT-PCR was conducted with primers and probes targeting at the N, ORF1a/b genes and a positive reference gene. The reaction system and amplification conditions were performed according to the manufacturer’s specification. The detection limit of cycle threshold (Ct) was set at 40 (500 copies/ml), with specimens having a Ct less than 40 considered positive. The cutoff Ct value of 40 was determined via the receiver operating characteristics (ROC) curve method. All procedures were performed under strict biosafety conditions and standard operating guidelines.

### 2.4 Statistics analysis

In this study, continuous variables were presented as median (P_25_, P_75_), while categorical variables were summarized as the counts and percentages. The Mann–Whitney U tests and Kruskal–Wallis tests were applied to continuous variables as appropriate, chi-square test or Fisher’s exact test were appropriately applied to categorical variables. *P-value* less than 0.05 was considered statistically significant. Statistical analysis was performed with IBM SPSS Statistics 25 and graphic representations were performed with GraphPad Prism 9.5.1 software.

## 3 Results

### 3.1 Study cohorts

**Figure 1:**
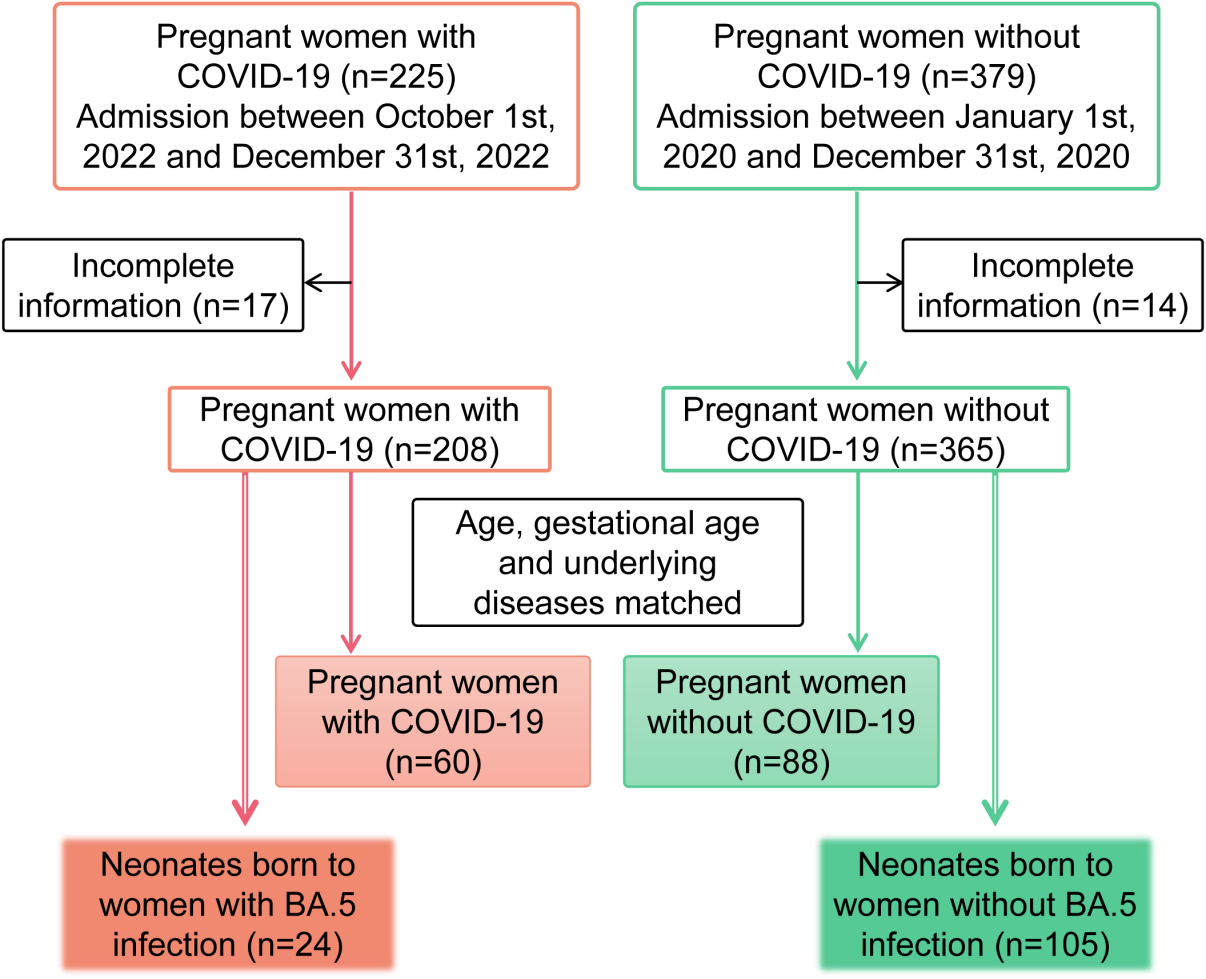
Flow chart of study cohorts. Case number, inclusion and exclusion criteria were labeled. Patients included in the analysis are highlighted.

Since October 2022, BA.5.2 and its sub-lineages became widespread in Guangzhou (China CDC weekly, 2023). The primary objective of this study was to investigate the potential differences in maternal, perinatal and neonatal outcomes associated with Omicron BA.5 infection. There were 225 pregnant women admitted in Guangzhou Eighth Hospital, Guangzhou Medical University with SARS-CoV-2 infection between October 1st, 2022 and December 31st, 2022. As a control, another 379 pregnant women without COVID-19 admitted between January 1st, 2020 and December 31st, 2020 were included. Excluding those with incomplete information, there were 208 pregnant women with COVID-19 and 365 non-infected controls. The infection status of all subjects was verified by viral RNA testing. Among the pregnant women with COVID-19, 91.8% (191/208) had received at least one dose of the inactivated COVID-19 vaccine. To facilitate a more accurate comparison of biochemical indicators, we matched the two groups based on age, gestational age, and underlying diseases, ultimately selecting 60 pregnant women with COVID-19 and 88 non-infected pregnant women for comparative analysis. Additionally, 24 neonates were delivered by 24 mothers infected with Omicron BA.5 during the admission. To further assess the impact of BA.5 infection on neonatal health, a comparison group consisting of 105 neonates born to 105 mothers without COVID-19 was also included in the study. The participant flow chart is shown in **Figure 1**.

### 3.2 Clinical characteristics of pregnant women with Omicron BA.5 infection

First, we analyzed the basic demographic and etiological characteristics of pregnant women infected with Omicron BA.5. The International Federation of Gynecology and Obstetrics (FIGO) defined advanced maternal age (AMA) as maternal age≥35 years at the time of delivery. AMA is associated with increased risks of severe COVID-19 outcomes and higher maternal and pregnancy complication rate (Allotey et al., 2020; Nana and Nelson-Piercy, 2021; Shams et al., 2022). In this study, the infected pregnant women were divided into AMA (≥35 years) and younger groups to assess whether AMA exacerbates Omicron BA.5 infection (**Table 1**).

**Table 1:** Clinical characteristics of pregnant women infected with Omicron BA.5.

Characteristics	Pregnant (n=208)		<35y (n=167, 80.29%)		≥0.2 (n=41, 19.71%)		*P*
Age (years), median(P_25_-P_75_)							
	30 (26.75-33)		29 (26-31)		36 (36-39)		
BMI, n(%)							
<25	148	(71.15)	121	(72.46)	27	(65.85)	0.159
25-29	51	(24.52)	41	(24.55)	10	(24.39)	
≥24	9	(4.33)	5	(2.99)	4	(9.76)	
Gestation at symptom onset, n(%)							
<13 weeks	63	(30.29)	51	(30.54)	12	(29.27)	0.957
14-28 weeks	72	(34.61)	57	(34.13)	15	(36.58)	
>28 weeks	73	(35.10)	59	(35.33)	14	(34.15)	
Vaccination, n(%)							
0-dose	17	(8.17)	14	(8.38)	3	(7.32)	0.078
1-dose	9	(4.33)	8	(4.79)	1	(2.44)	<0.001
2-dose	77	(37.02)	68	(40.72)	9	(21.95)	
3-dose	105	(50.48)	77	(46.11)	28	(68.29)	
Classification, n (%)							
Asymptomatic	14	(6.73)	8	(4.79)	6	(14.63)	0.064
Mild	192	(92.31)	157	(94.01)	35	(85.37)	
Moderate	2	(0.96)	2	(1.20)	0	(0.00)	
Comorbidity, n(%)							
No	189	(90.87)	154	(92.21)	35	(85.36)	
Yes	19	(9.13)	13	(7.79)	6	(14.64)	
Hypertension	3	(1.44)	2	(1.20)	1	(2.44)	>0.999
Diabetes	15	(7.21)	10	(5.99)	5	(12.2)	0.298
Psychosis	0	(0.00)	0	(0.00)	0	(0.00)	–
Thyroid	1	(0.48)	1	(0.6)	0	(0.00)	>0.999
Symptoms, n(%)							
Rhinocleisis/nasal discharge	39	(20.10)	32	(20.13)	7	(20.00)	>0.999
Fever (Tver9)℃)	115	(59.28)	94	(59.12)	21	(60.00)	>0.999
Cough	126	(64.95)	106	(66.67)	20	(57.14)	0.382
Expectoration	67	(34.54)	55	(34.59)	12	(34.29)	>0.999
Throat discomfort	70	(36.08)	57	(35.85)	13	(37.14)	>0.999
Chest distress/anhelation	4	(2.06)	4	(2.52)	0	(0.00)	0.771
Nausea and vomiting	10	(5.15)	10	(6.29)	0	(0.00)	0.271
Headache	25	(12.89)	20	(12.58)	5	(14.29)	>0.999
Muscle or joint pain	36	(18.56)	29	(18.24)	7	(20.00)	0.998
Tiredness	45	(23.20)	37	(23.27)	8	(22.86)	>0.999
Hyposmia	2	(1.03)	2	(1.26)	0	(0.00)	>0.999
Diarrhea	3	(1.55)	2	(1.26)	1	(2.86)	>0.999
Celialgia	40	(20.62)	30	(18.87)	10	(28.57)	0.292
Uterine contraction	18	(9.28)	15	(9.43)	3	(8.57)	>0.999
Uterine bleeding	7	(3.61)	6	(3.77)	1	(2.86)	>0.999

**Figure 2:**
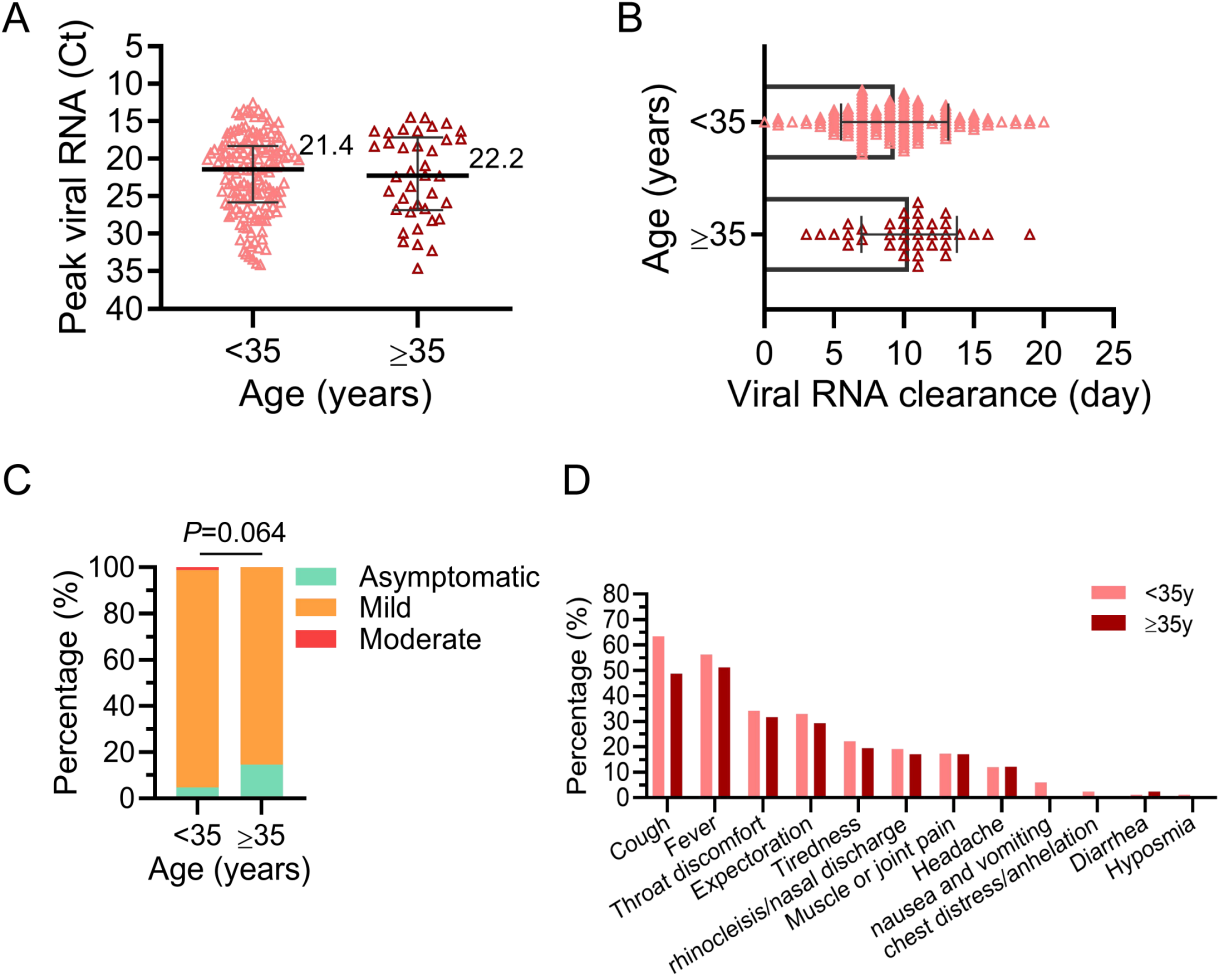
Etiologic features of pregnant women infected with Omicron BA.5. The infected women were divided into two groups: advanced maternal age (AMA, ≥35y) and younger (<35y). **(A)** Peak viral RNA (Ct value) in the upper respiratory tract of infected pregnant women. The medians are shown in the figure. **(B)** The duration of positive upper respiratory viral RNA in infected pregnant women. **(C)** The proportion of different disease severity after infection in the two groups. Chi-square test was used for analysis. **(D)** Prevalence of symptoms reported by pregnant women during infection. No P-value was marked if there was no statistical difference.

On the whole, however, no statistically significant differences were observed between these two groups. The peak viral titers in nasopharyngeal or oropharyngeal swabs were comparable for pregnant women under 35 (median Ct=21.4) and those over 35 (median Ct=22.2), with no significant difference (**Figure 2A**). Similarly, the time for viral RNA to turn negative was similar between these two groups (<35y, 9.3 ± 3.8 days; ≥35y, 10.4 ± 3.4 days, *P*=0.142, **Figure 2B**). The majority of cases in both groups were mild (<35y, 94.01%; ≥35y, 85.37%) or asymptomatic (<35y, 4.79%; ≥35y, 14.63%, **Figure 2C**). Fortunately, no severe or critical cases were found judging in these infected pregnant women from the clinical signs and symptoms. Moreover, the most prevalent symptoms during BA.5 infection in pregnant women were cough (64.95%), fever (59.28%) and throat discomfort (36.08%) (**Figure 2D**), aligning with symptoms observed in the general population. It is worth mentioning that 91.8% (191/208) of the pregnant women with COVID-19 in this study had been vaccinated against SARS-CoV-2 Vaccination coverage with three doses was significantly higher among pregnant women of AMA compared to the younger group (**Table 1**).

### 3.3 Changes of hematologic and biochemical data in pregnant women infected with Omicron BA.5

**Figure 3:**
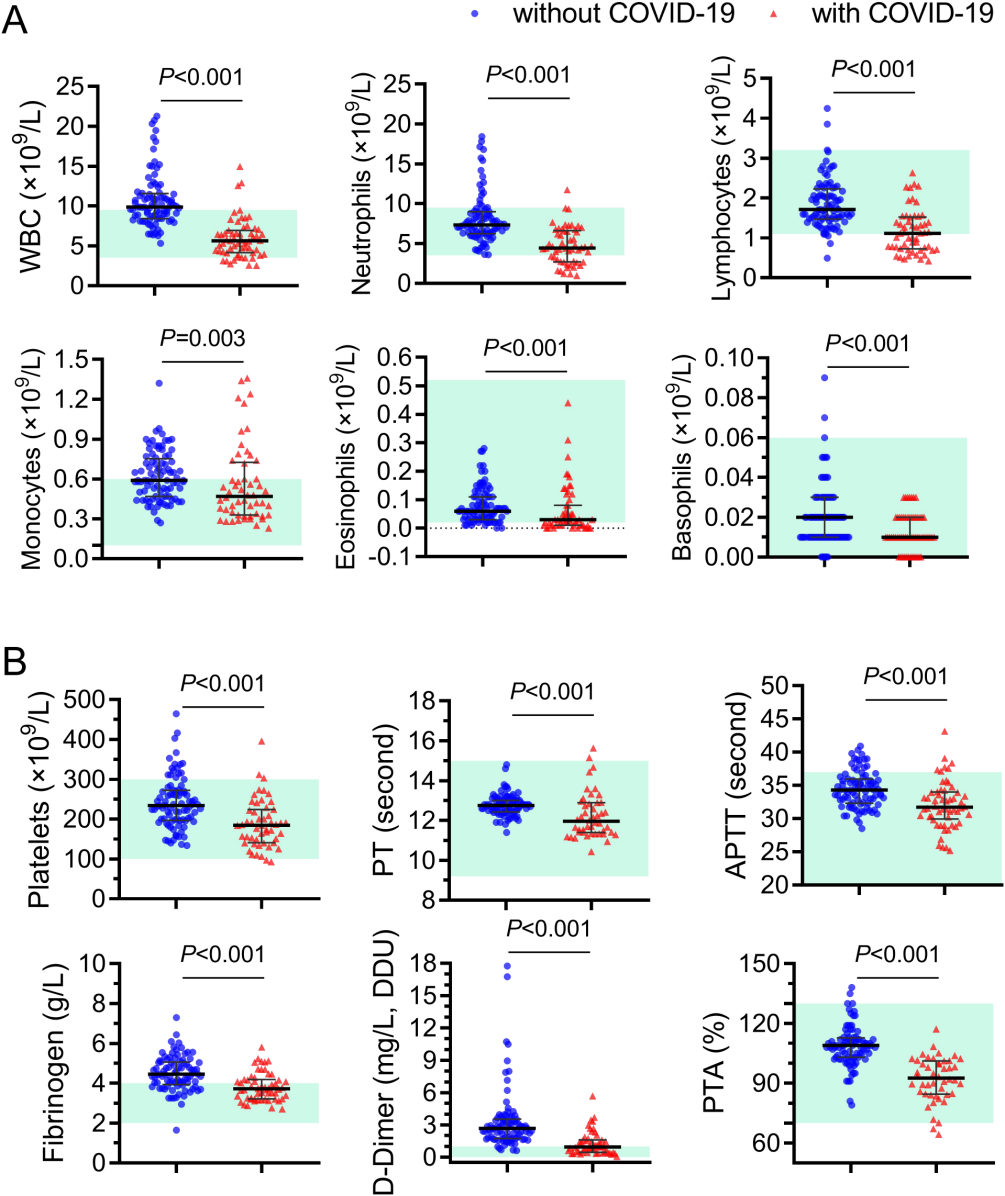
Different blood-cell counts and coagulation function indicators in pregnant women with and without Omicron BA.5 infection. **(A)** Different white blood cell counts. **(B)** Comparison of coagulation function indicators. Red triangels and blue circles represent pregnant women with and without COVID-19, represently. WBC, white blood cells; PT, prothrombin time; APTT, activated partial thromboplastin time; PTA, prothrombin activity.

Next, a thorough analysis was conducted to assess the changes of hematologic and biochemical indicators in pregnant women infected with BA.5 (**Figure 3**). Pregnant women with and without COVID-19 were carefully matched in terms of age, gestational age and underlying diseases. The results of blood routine examination indicated that the white blood cell count of pregnant women with BA.5 infection was significantly lower than that of pregnant women without COVID-19 (median 5.64×10^9^/L Vs. 9.87×10^9^/L, *P*<0.001), especially the number of neutrophils and lymphocytes showed the greatest change (**Figure 3A**). The group with COVID-19 showed lower hemoglobin levels, but the results in both groups were within the normal range (median 109 g/L Vs. 115 g/L, *P*=0.027, **Supplementary Table 1**).

Both COVID-19 and pregnancy are associated with thrombosis (Hwang et al., 2023). Normal pregnancy is accompanied by increased coagulation factors such as fibrinogen (James, 2009). Multiple meta-analyses studies report high rates of coagulopathy and thrombosis across multiple tissues and organ sites among patients infected with COVID-19 since early in the pandemic (Malas et al., 2020; Tan et al., 2021). Initially, it was hypothesized that Omicron BA.5 infection might potentially exacerbate the hypercoagulable state in pregnant women. However, when compared with the control group, pregnant women infected with BA.5 exhibited significantly (*P*<0.001) lower values for platelet (185×10^9^/L Vs. 234×10^9^/L), prothrombin time (PT, 12.75s Vs. 11.97s), activated partial thromboplastin time (APTT, 34.30s Vs. 31.71s), fibrinogen (4.45g/L Vs. 3.72g/L), D-Dimer (2.67ng/L Vs. 0.96ng/L) and prothrombin activity (PTA, 109.0% Vs. 92.7%) than those non-infected controls (**Figure 3B**). In addition, the indicator values in most of pregnant women infected with BA.5 are within the normal reference value range. These results indicated that infection with BA.5 did not worsen blood clotting function in these pregnant women.

### 3.4 Impact of Omicron BA.5 infection on maternal outcomes in pregnant women

Among the pregnant women infected with BA.5, there were 24 mothers gave birth to 24 neonates during the admission. To explore the impact of SARS-CoV-2 BA.5 infection on maternal perinatal and neonatal outcomes during pregnancy, we matched 105 pregnant women without Omicron BA.5 infection regarding age, gestational age and underlying diseases. 105 neonates born to these mothers were included (**Table 2**). Comparable levels of maternal age, reproductive history, BMI and comorbidity rates were found between the two groups. Though the gestational age at delivery of women with COVID-19 showed a litteyounger [38 (36-39) weeks] than that of women without COVID-19 [38 (38-39) weeks, *P*=0.049], nearly 80% (19/24) of the pregnant women with COVID-19 gave birth to full term. In terms of pregnancy risk, three of the 24 pregnant women with COVID-19 were assessed as having a risk of infectious disease plus extremely high risk of childbirth; four cases were assessed as infectious diseases with relatively high risk according to the Implementation Measures for Pregnancy Risk Assessment and Management of Pregnant Women in Guangzhou (**Table 2**) (https://www.gz.gov.cn/gfxwj/sbmgfxwj/gzswsjkwyh/content/mpost_5488994.html).

**Table 2:** Maternal and neonatal outcomes between pregnant women with and without BA.5 infection.

	Puerpera with COVID-19 (n=24)	Puerpera without COVID-19 (n=105)	*P*
Maternal outcomes			
Maternal age, median(P_25_-P_75_), years	31(28.80-33.00)	30(27-33)	0.095
Reproductive history			
G	3 (2-4)	2 (1-4)	0.434
P	1 (0-1)	1 (0-1)	0.843
BMI (kg/m2)	25.4 (24.10-26.80)	26.5 (24.80-28.50)	0.070
Comorbidity, n(%)			
Hypertension	3 (12.50)	3 (2.90)	0.078
Diabetes	2 (8.30)	13 (12.40)	0.736
Pregnancy risk ^&^			
Green (low)	0 (0)	35 (33.30)	–
Yellow (moderate)	0 (0)	2 (1.90)	–
Orange (relatively high)	0 (0)	35 (33.30)	–
Pink (high)	0 (0)	29 (27.60)	–
Red (extremely high)	0 (0)	3 (2.90)	–
Purple (with infectious diseases)	17 (70.80)	0 (0)	–
Purple + orange	4 (16.70)	1 (1.00)	–
Purple+red	3 (12.50)	0 (0)	–
Prenatal symptoms			
Celialgia	15 (62.50)	55 (52.40)	0.369
Uterine contraction	18 (75.00)	59 (56.20)	0.090
Uterine bleeding	2 (8.30)	32 (30.50)	0.038
Delivery approaches			
Cesarean delivery	16(66.67)	56 (53.33)	0.235
Vaginal delivery	8(33.33)	49 (46.67)	
Delivery status			
Intrapartum hemorrhage-cesarean section	300 (200-300)	–	–
Cesarean section Operation duration (min)	58 (50.75-71.50)	–	–
Intrapartum hemorrhage-eutocia	150 (100-150)	–	–
Intrapartum hemorrhage-all	200 (150-300)	–	–
Postpartum symptom			
Abnormal postpartum hemorrhage	0 (0)	–	–
Abnormal prenatal abdominal pain	0 (0)	–	–
Abnormal postpartum vaginal bleeding	0 (0)	–	–
Discharge status			
Steady	24 (100)	105 (100)	–
Admitted to ICU	24 (100)	–	–
Neonatal outcomes			
Gestational age at delivery, weeks	38(36-39)	38(38-39)	0.049
Full-term	19 (79.20)	92 (87.60) 13 (12.4) 2 (1.9) 105 (100)	0.281
Premature delivery	5 (20.80)	13 (12.40) 2 (1.9) 105 (100)	
Intrauterine hypoxia	3 (12.50)	2 (1.90) 2 (1.9) 105 (100)	0.015
Oxygen therapy	24 (100)	105 (100) 13 (12.4) 2 (1.9) 105 (100)	>0.999
Amniotic fluid			
Amniotic fluid clear	16	–	–
amniotic fluid turbidity I°	1	–	–
amniotic fluid turbidity III°	2	–	–
Birth weight, kg	3(2.70-3.33)	3.1(2.80-3.40)	<0.001
Apgar score			
Apgar 1min	10(9-10)	10(10-10)	–
Apgar 5min	10(10-10)	10(10-10)	–
Apgar 10min	10(10-10)	10(10-10)	–
Postpartum conditions			
breastfeeding	0	–	–
Fever	0	–	–

&: This is the pregnancy risk grading system implemented in Guangzhou for pregnant women. It grades pregnant women based on their basic conditions. Green indicates a favorable condition with a low pregnancy risk; yellow indicates the presence of certain risk factors and a moderate risk; orange indicates a certain threat to the safety of the mother and baby, with a relatively high risk; pink indicates a significant threat to the safety of the mother and baby, with a high risk; red indicates that continuing the pregnancy may endanger the life of the pregnant woman, with an extremely high risk; and purple indicates that the pregnancy is complicated by infectious diseases.

As for prenatal symptoms, 8.3% (2/24) of women with COVID-19 experienced uterine bleeding, significantly lower than that of women without COVID-19 (30.5%, 32/105, *P*=0.038). None of women with COVID-19 experienced abnormal postpartum hemorrhage, abdominal pain, or vaginal bleeding in this study. The vaginal delivery and cesarean section rates were comparable between women with and without SARS-CoV-2 BA.5 infection (33.3% vs. 46.7%, *P*=0.235; 66.7% vs. 53.3%, *P*=0.235). These results show that BA.5 infection has relatively little effect on the pregnancy status of pregnant women.

### 3.5 Impact of Omicron BA.5 infection during pregnancy on neonatal outcomes

On the other hand, we also analyzed the impact of maternal COVID-19 infection on neonatal outcomes. All of the 24 neonates born from women with COVID-19 in this study were alive at the moment of birth (**Table 2**). Among them, 8 (33.3%) neonates were born via vaginal delivery, whereas 16 (66.7%) were born by cesarean delivery, similar with those neonates born from women without COVID-19. However, neonates born to infected mothers had a higher rate of intrauterine hypoxia than those born to mothers without SARS-CoV-2 infection (12.5% Vs. 1.9%, *P*=0.015). Moreover, neonates born to infected mothers had a slightly lower birth weight (median 3kg vs. 3.1kg, *P*<0.001).

During the Omicron wave, neonates of unvaccinated mothers experienced significantly higher risks of adverse outcomes compared to infants born to vaccinated mothers (Barros et al., 2024). Among 24 deliveries in this cohort, all 9 neonates born to mothers with three doses of inactivated vaccine were full-term, while 3 of 6 neonates from unvaccinated or partially vaccinated mothers were preterm (**Supplementary Table 2**). Due to the small sample size, these findings are descriptive and not statistically compared.

**Figure 4:**
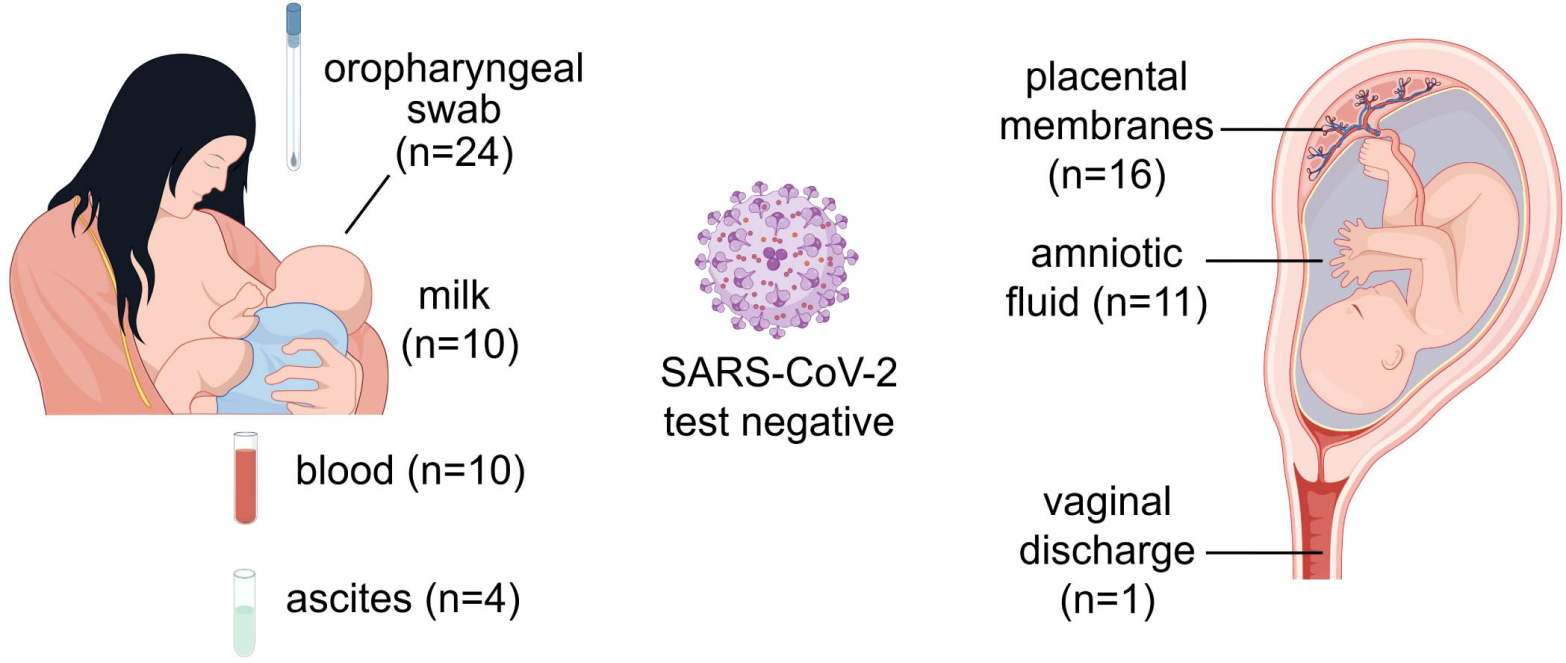
Nucleic acid test for SARS-CoV-2 from different samples of puerperae and neonates. The source of samples and the number of cases were marked on the graph. All samples were collected from puerperae except the oropharyngeal swabs. This figure was drawn by Figdraw.

Furthermore, to determine whether BA.5 infection can be transmitted to neonates through vertical transmission, we collected a series of specimens from these 24 puerperae with BA.5 infection and their newborns for SARS-CoV-2 nucleic acid test. To investigate potential vertical transmission of Omicron BA.5 variant, we collected amniotic fluid, placental membranes, and vaginal swabs during delivery. Furthermore, to assess the safety of breastfeeding in infected mothers, paired maternal breast milk and venous blood specimens were obtained within 24 hours postpartum for virological analysis. Additionally, based on previous reports of SARS-CoV-2 detection in peritoneal fluid (Coccolini et al., 2020), we collected four maternal ascites specimens during the delivery process for subsequent examination. All the specimens were negative for SARS-CoV-2 nucleic acid including 16 placental membranes, 11 amniotic fluid, 1 vaginal discharge, 10 breast milk, 10 venous blood and 4 ascites from pregnant women (**Figure 4**). No SARS-CoV-2 nucleic acid was detected in oropharyngeal specimens from all 24 neonates within 24 hours after birth. Overall, our results did not find SARS-CoV-2 is transmitted from puerpera to neonates via placenta or uterus.

## 4 Discussion

This study delineates the clinical characteristics and perinatal outcomes of pregnant women infected with Omicron BA.5 variant. Key findings indicate generally benign maternal outcomes following Omicron BA.5 infection, with no vertical transmission observed in neonates of infected mothers. Notably, 91.8% (191/208) of the infected pregnant women had received at least one dose of inactivated SARS-CoV-2 vaccine before infection. However, neonates of infected mothers exhibited a higher incidence of intrauterine hypoxia compared to controls (12.5% Vs. 1.9%, P=0.015). These findings suggest that, similar to non-pregnant adults, Omicron is less likely to cause severe COVID-19 in pregnant women and their neonates (Kerr et al., 2022). Nevertheless, enhanced prenatal care for pregnant women with COVID-19 remains essential to mitigate adverse neonatal outcomes.

As the virus continues to mutate, the effects of different SARS-CoV-2 strains on pregnancy vary. More severe maternal infection and worse pregnancy outcomes were observed during epidemics of Alpha and Delta variants than during epidemics of wildtype strain in UK and America (Strid et al., 2022; Vousden et al., 2022), especially among populations with low vaccination rates (Adhikari et al., 2022). Though increasing positivity rate was seen during Omicron variant predominance (Eid et al., 2023), SARS-CoV-2 infection in pregnancy during the Omicron-dominant wave was associated with reduced risk of complications than that during the Delta-dominant period, with decreased risk for a preterm birth or maternal critical care admission (Stock et al., 2022; Seaton et al., 2023). In addition, Omicron dominance resulted in lower rates of severe maternal and neonatal adverse outcomes when compared to waves of Beta and Delta variants (Mndala et al., 2022). It is reported that mild cases account for 79.8% to 97.4% in pregnant women with COVID-19 (Samadi et al., 2021; Arakaki et al., 2022; Kumari et al., 2022; Li et al., 2023). Consistent with these, 99.04% of pregnant women with Omicron BA.5 infection presented asymptomatic/mild disease, primarily manifesting influenza-like symptoms (cough, 64.95%; fever, 59.28%; throat discomfort, 36.08%) in this study. Nevertheless, some studies on pregnant women infected with Omicron have also indicated the presence of severe cases (Villar et al., 2023; Barros et al., 2024). Our finding may be related, to some extent, to the vaccination status of pregnant women. In our cohort of infected pregnant women, 87.5% (182/208) had received at least two doses of inactivated vaccine, which can protect against severe pregnancy and neonatal outcomes (Serra et al., 2022). Non-vaccination is associated with an increased risk of maternal morbidity and severe complications during Omicron infection (Villar et al., 2023).

Advanced maternal age (AMA, ≥35 years) is associated with increased risks of severe COVID-19 outcomes and higher maternal and pregnancy complication rate (Allotey et al., 2020; Nana and Nelson-Piercy, 2021; Shams et al., 2022). Notably, AMA did not correlate with increased disease severity or adverse outcomes in this cohort. This may be attributed to the widespread vaccination coverage and the weakened pathogenicity of Omicron variants, both of which enhance the protection of pregnant women. Notably, vaccination coverage with three doses was significantly higher among pregnant women of AMA compared to those younger. Further research is warranted to elucidate the underlying mechanisms linking AMA and Omicron infection outcomes.

Hematologic analysis revealed decreased leukocyte counts (particularly neutrophils/lymphocytes) in infected women, yet coagulation profiles (platelet, PT, APTT, fibrinogen, D-dimer and PTA) remained within normal ranges. This contrasts with early pandemic reports that pregnant women with COVID-19 are more likely to experience coagulopathy and venous thromboembolism in pregnant women (Servante et al., 2021; Zhou et al., 2021), aligning instead with recent evidence of low thrombotic risk from multinational cohort study or outpatient pregnant women with COVID-19 (Bikdeli et al., 2024; Othman et al., 2024). Moreover, in this study, the infected women had a relatively low rate of prenatal uterine bleeding, and no infected women experienced abnormal postpartum hemorrhage or vaginal bleeding. Notably, a subset of pregnant women without COVID-19 were admitted for conditions such as vaginal bleeding or childbirth preparation, which may explain the relatively higher incidence of prenatal hemorrhage observed in the non-infected group. Antepartum bleeding was considered to occur infrequently, and its incidence did not differ from illness severity or the pandemic wave and no association was demonstrated between isolated coagulation abnormalities and bleeding risk (Othman et al., 2024). The reported conclusions on the impact of Omicron infection on postpartum hemorrhage are inconsistent, which may be attributed to the varying contexts of the studies conducted (Li et al., 2023; Wang et al., 2024). In the present study, no instances of abnormal postpartum bleeding were observed among parturients infected with the Omicron BA.5 variant. However, given the limited sample size of the overall research, further studies are warranted to establish definitive findings.

During the epidemic of SARS-CoV-2 wildtype strain, a prospective cohort study in New York reported that COVID-19 could increase cesarean delivery rates and frequency of maternal complications in pregnant women (Prabhu et al., 2020). In our study, the rates of cesarean and premature delivery were comparable between women with and without SARS-CoV-2 BA.5 infection, which is consistent with previous reports during pre-Omicron variant predominance (Capretti et al., 2022). In addition to the weakened pathogenicity of Omicron variants, this may also be attributed to the fact that the majority of these pregnant women were vaccinated. Maternal vaccination can protect neonates from infection, and booster doses can reduce risks such as preterm birth (Villar et al., 2023; Barros et al., 2024). Administration of mRNA vaccines during pregnancy can reduce the risk of Omicron infection in infants aged six months and below. Additionally, pregnant women should not rely solely on pre-pregnancy vaccination. Completing the initial COVID-19 vaccination at any stage of pregnancy can delay infection and maintain effective neutralizing activity (Goh et al., 2023; Mahyuddin et al., 2024).

Moreover, there have been some reports of stillbirths and late miscarriages following SARS-CoV-2 infection. In our study, all of the 24 neonates born from women with BA.5 infection were alive at the moment of birth. Although the risk of vertical transmission of BA.5 infection is low, our findings indicate that newborns of infected mothers had a higher rate of intrauterine hypoxia than those controls, indicating that maternal infection will affect the status of fetus to a certain extent. A key contributing factor to prolonged hospitalization durations among parturients infected with the SARS-CoV-2 Omicron variant and their neonates appears to be fetal distress-a condition characterized by acute or chronic intrauterine hypoxia afflicting the developing fetus (Wang et al., 2024). This observation aligns with recent empirical evidence demonstrating that Omicron-infected gravidae exhibit markedly elevated rates of cesarean delivery and fetal distress relative to their non-infected counterparts (Li et al., 2023). Notably, there was a reduction in the risk of fetal distress and maternal referral among vaccinated women (Villar et al., 2023).Prenatal care, along with vaccination, should still be strengthened for pregnant women with COVID-19 to mitigate potential adverse neonatal outcomes.

Previous studies have confirmed the vertical transmission of SARS-CoV-2, although this is rare (Allotey et al., 2022). Numerous studies have reported that the positive rate for SARS-CoV-2 nucleic acid test by nasopharyngeal swab in infants ranging from 0.9 to 2.8%. (Woodworth et al., 2020; Flaherman et al., 2021; Kotlyar et al., 2021; Norman et al., 2021). Adhikari et al.’s recent study found that 3.1% of infants born to COVID-19-positive mothers tested positive for SARS-CoV-2 across the pre-Delta, Delta, and Omicron periods, and neonatal positivity rates remained consistent throughout these periods (Metz et al., 2022). In previous reports, the virus and viral fragments have been detected in maternal blood, placenta, amniotic fluid and breast milk. In this study, none of the 24 neonates was infected through vertical transmission. All the specimens collected, including placental membranes, amniotic fluid, breast milk, venous blood, ascites and vaginal secretions from mothers and oropharyngeal swabs from neonates, were negative for SARS-CoV-2 nucleic acid. Consistent with other studies, only rare case reports were with probable vertical transmission during COVID-19 (Hosier et al., 2020; Kirtsman et al., 2020; Zeng et al., 2020). On the other hand, the protective effect of vaccination on neonatal test positivity may also play a significant role during Omicron epidemic (Barros et al., 2024). However, it is required to confirm whether the transmission was vertical or due to environmental exposure after birth.

Among symptomatic and unvaccinated pregnant women, Omicron infection was associated with an increased risk of maternal morbidity and severe complications than that without COVID-19 (Villar et al., 2023). However, vaccination may prevent severe symptoms and complications derived from SARS-Cov-2 infection including maternal and neonatal fatalities as well as ICU admission (Carlsen et al., 2022; Dominguez-Ramirez et al., 2022; Male, 2022; Serra et al., 2022). Real-world evidence points to the good safety and effectiveness of the COVID-19 vaccine for pregnant women, including mRNA vaccine (Kharbanda et al., 2021; Shimabukuro et al., 2021; Wu et al., 2023). Neonates born to vaccinated mothers had lower risks of a positive test for SARS-CoV-2 during the first 4 months of life compared to mothers without vaccination (Carlsen et al., 2022). Considering the epidemiological situation in China, expert opinions emphasizes that pregnant women who are at risk of COVID-19 should be recommended to receive a full-dose COVID-19 vaccination (Yun et al., 2020). Widespread vaccination and past infection have established effective immunity to prevent severe outcomes in pregnant women after infection. Therefore, it is unlikely to observe more cases of maternal or fetal deaths due to COVID-19 infections in the future. Universal vaccination, comprehensive prenatal surveillance, and timely therapeutic intervention constitute critical strategies for the prevention and management of COVID-19 in pregnant populations.

There are some limitations in this cohort study. Firstly, the control group we selected consists of pregnant women visited the hospital before the outbreak of COVID-19, rather than pregnant women without COVID-19 during the same period, which might introduce some biases in the results. However, due to China’s management policies during the COVID-19 pandemic, hospitals designated to treat COVID-19 like our hospital couldn’t access non-infected pregnant women at the same time. But at least, our cohort ensured that the medical staff treating these two groups was relatively consistent. Secondly, as this is a retrospective study, our analysis is limited to the available data which may potentially constrain the statistical power of our findings. Future investigations employing more rigorously balanced sample designs are warranted to validate and extend these findings. The findings might not be entirely representative of the overall population but do reflect the characteristics of pregnant women infected with the BA.5 variant in Guangzhou region. The results provide additional insight into the clinical characteristics of pregnant women infected with the Omicron variant and the maternal and neonatal outcomes.

In summary, this study demonstrates favorable maternal and neonatal outcomes in vaccinated pregnant women with Omicron BA.5 infection following timely medical intervention. The neonates born to infected mothers have extremely low risks of vertical transmission. Universal vaccination, prenatal monitoring and prompt treatment are keys to preventing and treating COVID-19 during pregnancy. Further study is needed to explore whether maternal SARS-CoV-2 infection is associated with long-term maternal or neonatal health.

## Data Availability

The raw data supporting the conclusions of this article will be made available by the authors, without undue reservation.
