# Supplementary material for: Impact of Omicron BA.5 infection on maternal and neonatal outcomes
Source: Front Cell Infect Microbiol. 2025 May 2;15:1551602. doi: 10.3389/fcimb.2025.1551602 (PMC12082113; doi:10.3389/fcimb.2025.1551602)
Supplement: Supplementary file 1 [file Table1.docx]

**Supplementary Table 1** Hematologic and Biochemical data of pregnant women with and without COVID-19

| **Laboratory results** | **Pregnant with**  **COVID-19** | **n** | **Pregnant without COVID-19** | **n** | ***P*** |
| --- | --- | --- | --- | --- | --- |
| WBC | 5.64(4.195-6.765) | 59 | 9.87(8.435-11.51) | 88 | <0.001 |
| NEU | 4.435(2.74-6.5825) | 54 | 7.335(6.245-8.9825) | 88 | <0.001 |
| NEU% | 70.3(61.15-78.65) | 55 | 74.45(70.475-78.675) | 88 | 0.012 |
| BASO | 0.01(0.01-0.02) | 59 | 0.02(0.01-0.03) | 88 | 0.001 |
| BASO% | 0.2(0.1-0.3) | 59 | 0.2(0.1-0.3) | 88 | 0.691 |
| EOS | 0.03(0.01-0.08) | 59 | 0.06(0.03-0.11) | 88 | <0.001 |
| EOS% | 0.4(0.1-1.3) | 59 | 0.6(0.3-1.225) | 88 | 0.103 |
| LYM | 1.115(0.7375-1.4925) | 54 | 1.71(1.4775-2.19) | 88 | <0.001 |
| LYM% | 16.9(10.7-26.2) | 54 | 18.45(14.275-22.275) | 88 | 0.958 |
| MONO | 0.47(0.3325-0.69) | 54 | 0.59(0.47-0.7375) | 88 | 0.003 |
| MONO% | 8.4(7.1-9.875) | 54 | 6(4.9-6.9) | 88 | <0.001 |
| RBC | 4.005(3.7025-4.37) | 46 | 3.885(3.56-4.165) | 88 | 0.064 |
| CRP | 11.88(9.99-22.17) | 57 | 9.99(9.99-13.58) | 18 | 0.213 |
| Hct | 32.3(30.5-33.7) | 59 | 34.15(31.3-36.65) | 88 | 0.003 |
| HGB | 109(99.5-114.5) | 59 | 115(102.75-124) | 88 | 0.027 |
| MCV | 86.65(83-90.075) | 54 | 89.1(84.475-92.35) | 88 | 0.068 |
| MCH | 29.7(27.925-30.8) | 54 | 29.5(27.95-31.35) | 88 | 0.505 |
| MCHC | 338.5(330-344) | 54 | 334(328-341) | 88 | 0.044 |
| PLT | 185(145-223) | 51 | 234(198.25-270.75) | 88 | <0.001 |
| MPV | 9.85(9.025-10.875) | 54 | 10.1(9.375-11.025) | 88 | 0.378 |
| PT | 11.965(11.41-12.8775) | 46 | 12.75(12.475-13) | 88 | <0.001 |
| APTT | 31.705(30.13-33.96) | 56 | 34.3(32.375-35.85) | 88 | <0.001 |
| Fib | 3.715(3.2175-4.1675) | 56 | 4.45(3.9825-5.05) | 88 | <0.001 |
| PTA (%) | 92.655(84.72-100.91) | 46 | 109(103-112.25) | 88 | <0.001 |
| FDP | 6.6(4.155-9.555) | 55 | 4.48(3.29-5.9725) | 88 | 0.001 |
| INR | 1(0.955-1.03) | 55 | 0.95(0.94-0.98) | 88 | <0.001 |
| A/G | 1.39(1.295-1.49) | 50 | 1.35(1.23-1.5) | 88 | 0.323 |
| ALB | 34.2(32.7-35.6) | 50 | 35.9(34.175-37.725) | 88 | 0.003 |
| ALT | 9.54(7.21-12.68) | 50 | 8.5(6.475-11.825) | 88 | 0.437 |
| AST | 17.2(14.175-21.65) | 50 | 15.55(12.975-17.75) | 88 | 0.012 |
| AST/ALT | 1.83(1.465-2.3175) | 50 | 1.695(1.3775-2.12) | 88 | 0.146 |
| Cr | 50.45(44.975-54.125) | 50 | 43.35(39.675-50.725) | 88 | <0.001 |
| D Bili | 2.7(2.025-3.1) | 50 | 2.16(1.72-2.5725) | 88 | <0.001 |
| DB/TB | 0.58(0.49-0.66) | 50 | 0.52(0.4575-0.62) | 88 | 0.044 |
| GLB | 24.65(23.325-27.15) | 50 | 26.45(24.35-28.8) | 88 | 0.006 |
| Glu | 4.425(4.1275-4.8275) | 50 | 4.65(4.09-5.22) | 87 | 0.227 |
| I Bili | 1.805(1.1175-3.2875) | 50 | 1.935(1.3775-2.7675) | 88 | 0.828 |
| RDW-SD | 42.3(40-44.25) | 50 | 44.3(42.1-46.9) | 88 | <0.001 |
| PLT-PCT | 0.177(0.155-0.222) | 49 | 0.234(0.2-0.27925) | 88 | <0.001 |
| ADA | 9.035(7.845-10.5475) | 48 | 7.45(6.49-8.6475) | 88 | <0.001 |
| ChE | 4.935(4.45-5.465) | 48 | 5.98(5.32-6.62) | 88 | <0.001 |
| Ca2+ | 2.07(2.01-2.1025) | 48 | 2.17(2.11-2.245) | 87 | <0.001 |
| Cl- | 103.6(101.65-106.025) | 48 | 102.4(100.7-104.75) | 87 | 0.016 |
| I Phos | 1.16(1.1-1.2375) | 48 | 1.1(0.97-1.235) | 87 | 0.017 |
| K+ | 3.585(3.47-3.7) | 48 | 3.73(3.545-3.895) | 87 | 0.01 |
| LDH | 163(148.75-188.25) | 48 | 212.5(204.25-227.5) | 4 | 0.012 |
| Mg2+ | 0.74(0.7075-0.77) | 48 | 0.77(0.73-0.81) | 87 | 0.035 |
| Na+ | 136(134.5-138) | 47 | 137(136-138) | 87 | 0.075 |
| HBsAg | 0.02(0.02-0.02575) | 46 | 0.51(0.44-0.58) | 49 | <0.001 |
| PAB | 0.176(0.15425-0.19775) | 46 | 0.2155(0.18675-0.23625) | 88 | <0.001 |
| RBC | 4.005(3.7025-4.37) | 46 | 3.885(3.56-4.165) | 88 | 0.064 |
| RDW | 12.85(12.3-13.475) | 46 | 13.7(12.875-14.925) | 88 | <0.001 |
| UA | 260.9(220.4-299.5) | 46 | 303.4(260.55-371.225) | 88 | 0.001 |
| Urea | 2.255(1.8825-2.69) | 46 | 2.725(2.2-3.5) | 88 | <0.001 |
| Osm | 272(270-276) | 45 | 274(272-276) | 87 | 0.058 |
| T Bili | 4.62(3.4725-6.175) | 43 | 4.01(3.2675-5.3475) | 88 | 0.159 |
| T Chol | 4.505(3.615-5.4) | 42 | 5.76(5.11-5.8) | 3 | 0.187 |
| TRIG | 1.54(1.0075-2.0925) | 42 | 3.14(2.715-3.24) | 3 | 0.043 |
| CRY | 0(0-0.66) | 41 | 0(0-0) | 77 | 0.491 |
| T PROT | 62.7(58.2-67) | 41 | 62.8(59.9-65.55) | 88 | 0.97 |
| TT | 13.47(13.105-14.35) | 40 | 15.5(15.1-15.9) | 88 | <0.001 |
| PH | 6.5(6-6.5) | 38 | 6.5(6-6.5) | 81 | 0.688 |
| SPGR | 1.016(1.011-1.021) | 33 | 1.016(1.009-1.021) | 81 | 0.764 |
| TBA | 3.73(1.735-5.405) | 31 | 2.33(1.445-3.3275) | 88 | 0.041 |
| TP-Anti | 0.2875(0.28225-0.3) | 30 | 0(0-0.07) | 16 | <0.001 |
