# Supplementary material for: Impact of Omicron BA.5 infection on maternal and neonatal outcomes
Source: Front Cell Infect Microbiol. 2025 May 2;15:1551602. doi: 10.3389/fcimb.2025.1551602 (PMC12082113; doi:10.3389/fcimb.2025.1551602)
Supplement: Supplementary file 2 [file Table2.docx]

**Supplementary Table 2** The Impact of Vaccination in Pregnant Women on Their Clinical Classification and Neonatal Outcomes

| **COVID-19 Vaccine** | **0-1 dose**  **(n=26)** | | **2 doses**  **(n=77)** | | **3 doses**  **(n=105)** | |
| --- | --- | --- | --- | --- | --- | --- |
| **Classification, n (%)**  **Classification, n (%)**  (68.29) | | | | | | |
| Asymptomatic | 0 | (0.00) | 6 | (7.79) | 8 | (7.61) |
| Mild | 26 | (100) | 71 | (92.21) | 95 | (94.01) |
| Moderate | 0 | (0.00) | 0 | (0.00) | 2 | (1.20) |
| **Neonatal outcomes, n (%)** | n=6 | | n=7 | | n=9 | |
| Vaginal delivery | 1 | (16.67) | 1 | (14.29) | 5 | (55.56) |
| Cesarean delivery | 5 | (83.33) | 6 | (85.71) | 4 | (44.44) |
| Full-term  Premature delivery  Intrauterine hypoxia  Oxygen uptake | 3 | (50.00) | 6 | (85.71) | 9 | (100) |
| Premature delivery | 3 | (50.00) | 1 | (14.29) | 0 | (0.00) |
| Intrauterine hypoxia | 1 | (16.67) | 1 | (14.29) | 1 | (11.11) |
